# Genome Sequence of Classical Swine Fever Virus Genotype 1.1 with a Genetic Marker of Attenuation Detected in a Continuous Porcine Cell Line

**DOI:** 10.1128/genomeA.00375-15

**Published:** 2015-04-30

**Authors:** N. Tomar, A. Gupta, R. S. Arya, R. Somvanshi, V. Sharma, G. Saikumar

**Affiliations:** a Division of Pathology, Indian Veterinary Research Institute, Izatnagar, Uttar Pradesh, India; b Department of Veterinary Pathology, College of Veterinary Science, CAU, Aizawl, Mizoram, India; c Department of Bioscience & Biotechnology, Banasthali University, Rajasthan, India

## Abstract

The complete genome sequencing and analysis of a classical swine fever virus (CSFV) detected in a porcine kidney cell line revealed a close relationship with genotype 1.1 viruses circulating in India and China. The presence of consecutive T insertions in the 3′ untranslated region (UTR), as seen in vaccine strains of CSFV, suggested some degree of attenuation.

## GENOME ANNOUNCEMENT

Classical swine fever (CSF) is an Office International des Epizooties (OIE)-listed disease of pigs and wild boars having a serious economic impact on pig production in regions of endemicity. The causative agent, CSF virus (CSFV), belongs to the genus *Pestivirus* within the family *Flaviviridae*. The CSFV genome is a single positive-stranded RNA of approximately 12.3 kb containing an ~11.7-kb-long single open reading frame (ORF) flanked by the 5′ and 3′ untranslated regions (UTRs). The UTRs contain signals for viral replication, transcription, and translation. The large open reading frame typically encodes a polyprotein of 3,898 amino acids that undergoes co- and posttranslational processing by cellular and viral proteases to produce four structural and eight nonstructural proteins (1). CSFV has one serotype divided into three major genogroups, 1, 2, and 3, each comprising three to four subgenogroups (2).

We report here the complete genome sequence of CSFV (CSFV-PK15C-NG79-11), detected as an adventitious pestivirus while screening cell lines and tissue culture reagents before their use in the laboratory. Total RNA was extracted from a PK15 cell line using the TRIzol reagent, and cDNA was synthesized using SuperScript III reverse transcriptase (RT), according to the manufacturer’s instructions (Invitrogen, USA). The whole genome of CSFV-PK15C-NG79-11 was amplified as overlapping fragments by reverse transcription-PCR (RT-PCR) and directly sequenced in both directions on an ABI Prism 3500xL DNA sequencer. The sequences were edited and assembled to build the contig. Preliminary analysis was conducted by BLAST on the NCBI website, and the phylogenetic relationship with other CSFVs was ascertained by using MEGA 6 (3).

The complete genome of CSFV-PK15C-NG79-11 was determined to be 12,302 nucleotides (nt) long, including the 5′ UTR (373 nt) and 3′ UTR (232 nt) regions. The single large ORF (11,697 nt) was capable of coding for a polyprotein of 3,898 amino acids. This virus showed a maximum homology of 99% with an Indian isolate, CSFV/IVRI/VB-131 (accession no. KM262189), and 98% with some historical Chinese isolates, such as Shimen/HVRI (accession no. AY775178). The nucleotide sequence of CSFV-PK15C-NG79-11 shared 90 to 99% similarity with group 1 viruses, 85 to 86% with group 2 viruses, and 85% with group 3 viruses.

A comparison of the nucleotide sequences of the individual genes of the CSFV-PK15C-NG79-11 with those of CSFV/IVRI/VB-131 showed 99 to 100% homologies for all genes, except for 98% in 3′ UTR region. A similar analysis with Shimen/HVRI revealed 97% to 99% sequence homology. In comparison to Shimen/HVRI, the 3′ UTR of CSFV-PK15C-NG79-11 had a 6-nt insertion (TTTTTT) at position 12133 and deletion of 1 nt at position 12225. CSFV/IVRI/VB-131 had only a 4-nt (TTTT) insertion in this region. Attenuated vaccine strains carry a higher number of nucleotide insertions (4).

Phylogenetic analysis of CSFV-PK15C-NG79-11 showed that it belonged to genotype 1.1, sharing a close relationship with virulent viruses, such as Shimen/HVRI and CSFV-GZ-2009 from China. However, the 6-nt T insertion in the 3′ UTR of the CSFV-PK15C-NG79-11 suggested some degree of attenuation.

### Nucleotide sequence accession number.

The complete genome sequence of CSFV-PK15C-NG79-11 has been submitted to GenBank under the accession no. KC503764.
